# Using computer games to explore foraging-predation trade-offs and spatial learning in humans

**DOI:** 10.1098/rsos.260389

**Published:** 2026-09-09

**Authors:** Stav Subach, Arik Dorfman, Adi Bar, Tali Potudin, Aziz Subach, Inon Scharf

**Affiliations:** School of Zoology, George S Wise Faculty of Life Sciences, Tel Aviv University, Tel Aviv, Israel

**Keywords:** carryover effects, computer game, optimal foraging theory, personality, spatial learning, trade-off

## Abstract

Foraging requires organisms to balance energetic gains against multiple costs, including predation risk, while updating decisions as conditions change. Although these processes are well studied in animals, ecologically grounded paradigms for testing behavioural updating and cross-task generalization in humans remain limited. We developed two computer-game assays inspired by animal foraging research to examine how humans adjust behaviour under simulated predation risk, whether prior risk exposure affects later decisions and whether spatial performance generalizes across tasks. Participants completed a central-place open-field foraging task under safe and risky conditions and a maze-navigation task assessing spatial learning. Predation risk reduced foraging effort and increased hiding behaviour. Behavioural responses also depended on task order: individuals exposed to risk first showed attenuated shifts across contexts, indicating carryover effects and incomplete updating of environmental state. Spatial performance generalized across tasks under both safe and risky contexts. In the maze task, performance improved across trials with a negatively accelerated learning curve, consistent with spatial learning dynamics. These findings show that ecologically grounded computer games can reveal imperfect updating under changing risk while providing a controlled framework for studying human decision-making under ecological constraints.

## 1. Introduction

Classical foraging theory predicts that individuals should continue exploiting resources as long as energetic gains outweigh associated costs, including movement costs, missed-opportunity costs and predation risk [1–3]. Among these, predation risk is a particularly strong selective force shaping behaviour and life-history traits [4]. Predators often exert stronger indirect effects, through changes they induce in the behaviour of potential prey, than through direct mortality [5–7]. Foragers, in turn, must continuously balance resource acquisition against safety [8–10].

In addition to the food-safety trade-off, foragers face other ecological conflicts, particularly the exploration–exploitation dilemma, which requires balancing the search for new resources against the continued use of familiar ones [11,12]. Predation risk may shift this balance, favouring reduced movement and prolonged use of known patches [13]. Resolving such trade-offs lies at the core of decision ecology [14]. In turn, many optimal foraging models implicitly assume that individuals possess reliable knowledge of their environment, an assumption that depends on learning [15,16]. Yet adaptive responses depend not only on acquiring information, but also on updating behaviour when conditions change. Imperfect updating may therefore generate behavioural carryover, such that prior experience continues to shape decisions even after environmental risk shifts.

In dynamic environments, individuals are frequently exposed to changing risk or resource availability. Optimal models typically assume rapid adjustment to current conditions, yet behavioural responses may persist beyond the context in which they were acquired [17]. Such carryover effects, where prior exposure influences subsequent decisions even after environmental conditions change, may have important consequences for adaptive behaviour [18,19]. If individuals fail to update their assessment of risk, previous experience may shape later decisions even after conditions change. Recent human studies also support such carryover effects under changing environments (e.g. [20,21]), and recent ecologically inspired work further shows that human foraging under threat is shaped by individual differences and threat-related behavioural strategies [22]. A second question is whether spatial performance reflects stable individual differences that generalize across distinct tasks. Some studies report dissociations across cognitive tasks, whereas others find positive correlations and cross-context consistency [23–29]. Although foraging performance is often repeatable within contexts [30,31], it remains unclear to what extent such performance generalizes across structurally distinct ecological tasks. Here, we address both questions within ecologically structured foraging paradigms that integrate spatial structure and predation risk.

Learning enables individuals to improve performance with accumulating experience and to form internal representations of environmental structure [32,33]. In spatially structured environments, especially those encountered by central-place foragers, learning is essential because individuals must both locate resources and return to a home base [34,35]. Spatial learning has therefore been extensively studied in central-place foragers, including social hymenopterans, rodents and humans [36–38]. Learning dynamics is often characterized using learning curves, which describe improvements in performance across repeated trials [39,40]. These curves are typically negatively accelerated [41], although individual trajectories may vary substantially. As experience accumulates, the effective profitability of patches may change, suggesting that foraging outcomes depend not only on environmental conditions but also on the learner's stage of experience [15].

Although these questions have often been examined separately, few studies have tested carryover effects and cross-task performance generalization within the same individuals. Such within-individual approaches are important because they allow behavioural updating and generalization across contexts to be assessed under shared conditions. Computer-game paradigms provide a methodological bridge between animal foraging studies and human behavioural experiments by preserving functional ecological constraints while allowing experimental control and high-resolution spatial and temporal measurement [42,43]. They do so not by reproducing species-specific natural environments, but by creating functional analogues of shared decision problems, which can be compared across taxa.

Previous studies have shown that virtual tasks can reproduce key ecological dynamics, including risky foraging and predator–prey interactions [44–47]. Digital simulations have been used to model a range of ecological decision problems, including predator–prey interactions, visual and spatial foraging, patch exploitation, adaptive responses to uncertainty and the integration of asocial and social learning in collective foraging [48–51]. Game-based approaches have also been used to study decision processes in structured environments beyond ecological applications [52–54]. In this sense, computer games can serve not merely as analogies but as controlled model systems for testing ecological decision principles. However, most human foraging-like studies have focused on either risk-sensitive behaviour or search patterns within a single task. Our approach extends this work by combining risk manipulation, carryover effects and cross-task performance generalization within one integrated experimental system.

Here, we developed two controlled game-based paradigms inspired by animal foraging research: (i) an open-field central-place foraging task conducted under safe and risky contexts and (ii) a maze-navigation task assessing spatial learning. Using performance-based incentives and detailed spatial tracking, we examined whether individuals update foraging-like behaviour under predation risk and whether prior exposure to risk generates carryover effects across contexts. We also tested whether spatial performance generalizes across distinct but related tasks, which may indicate stable individual differences, and whether navigation performance improves with repeated experience. We predicted that predation risk would reduce foraging intensity and increase hiding behaviour, that prior exposure to risk would influence subsequent behaviour, that performance would improve with experience and that individuals would show correlated performance across tasks.

## 2. Materials and methods

The experiment consisted of two computer games: an open-field foraging game and a maze navigation game. The two tasks were selected because they represent experimental paradigms already used in animal research, while differing structurally and sharing core spatial decision demands. This allowed an initial test of whether performance generalizes across structurally distinct but related tasks. A total of 35 human participants voluntarily took part in the study, recruited through advertisements on student social networks. To motivate participants to perform at their best, a monetary reward was offered to the top scorer in each game, giving each participant the opportunity to earn two separate rewards. The reward structure was explained in advance, and prizes were distributed after all participants had completed the experiment. Both games recorded player coordinates every second and exported the data as CSV files. All behavioural measures were generated automatically by the computer game, such that data recording was blind to our expectations and did not involve any human observer. Because experimental conditions were embedded in the task design, data analysis could not be conducted blind to the experimental condition.

### 2.1. Open-field foraging game

**Figure 1.**
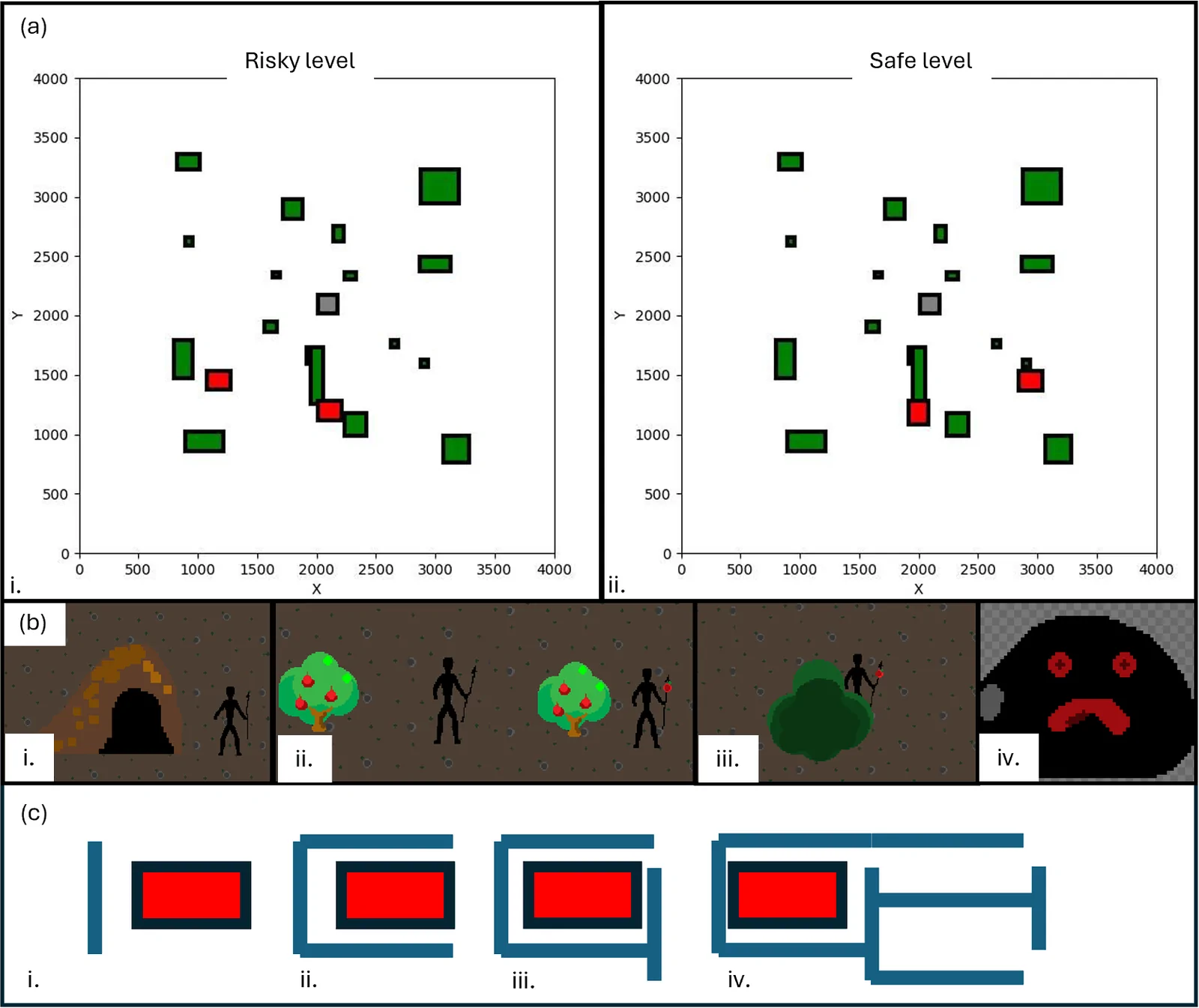
The open-field foraging game. (a) Maps of the risky and safe levels. The grey rectangle represents the central place, the green rectangles represent hiding spots and the red represent food patches. The x- and y-axes indicate the map limits in pixels. (b) The different game entities: (i) the central place and the player. (ii) The food patch before and after delivering a food item. (iii) The player/avatar is hiding in a bush. (iv) An enemy or predator. (c) Illustration of increasing barrier complexity around the first patch the player encountered. The red rectangle represents the food patch, and the blue lines represent the barriers. Each encounter generated another barrier. When players encountered the second patch, no barriers formed around it.

We developed a two-dimensional, top-down game, where the player views the action from above, in GameMaker [55], and designed it to simulate central-place foraging in an open-field environment (figure 1). The player sees an area equivalent to 600 pixels out of 3500 pixels (the whole map size) ahead. The game included two levels: a ‘risky’ level incorporating simulated predation risk and a ‘safe’ level without risk. Each level lasted five minutes and ended automatically. The game consisted of the following elements: (i) a player avatar: a stickman figure controlled using the arrow keys. (ii) A central place (‘a cave’): the player's starting location and the destination for depositing collected food items to score points. (iii) A scoreboard, which displayed the number of successful food deliveries to the central place. This was visible to participants but not used in the data analysis and is referred to as the ‘perceived score’. (iv) Two food patches, represented by fruit-tree icons. Contact with a patch caused the player avatar to collect a food item, and returning it to the central place increased the score by one. Patch locations differed between the risky and safe levels. (v) Enemies or predators, which were present only at the risky level. Whenever the player was farther than 350 pixels from the predator or was in a bush, the predator followed a predefined path in the shape of a spiral. When the player was nearby, the predator moved towards the player. Upon contact with the player, the predator disappeared and respawned at a random map location, while the player was immobilized for two seconds and lost one point from the perceived score. These elements were designed to induce perceived predation risk (‘fear effects’) while keeping resource availability constant. Although predator encounters imposed a brief time penalty, our primary outcome measures focused on behavioural allocation (e.g. hiding time and patch visitation) rather than on the on-screen score. This should mimic indirect predation effects, or more specifically, predator-model experiments in animal behaviour, in which predator models are used, leading to behavioural changes of potential prey [56–58]. Another reason that the perceived score was not used was that we tried to measure foraging effort rather than success. The perceived score reflects game-specific success (combining foraging and predator evasion), whereas our analyses targeted generalizable behavioural allocation patterns. However, the effort or time that individuals invest in foraging relative to antipredator activities, i.e. hiding or running away in the game, is general enough to be a widespread trade-off. While the temporal immobilization could slightly influence performance, its primary effect was psychological, reflecting indirect predation effects. (vi) Hiding places, represented by bushes. When the player entered a bush, predators stopped pursuing and resumed passive movement, as described above. However, collisions with predators still incurred penalties.

Participants received printed instructions before starting, including: (i) the goal: to collect as many food items as possible within five minutes while avoiding predators. (ii) How to control the player. (iii) How predators interact with the player and with the hiding places. (iv) The order in which the two levels, risky and safe, will be played. The order was designed to simulate natural environments where animals typically know whether they are foraging in a safe or risky time, such as nocturnal animals foraging on a full moon versus a moonless night [59].

To examine whether participants altered their foraging behaviour across the exploration–exploitation trade-off, we introduced a cost for repeatedly exploiting the first food patch discovered. After returning with the first food item, a barrier was placed at the first patch's location, increasing complexity with each of the next three visits (figure 1*c*). These penalties did not apply to the second patch. Thus, repeated visits to the first patch became progressively more costly, encouraging participants to abandon continued exploitation and explore the alternative patch. Participants were not informed in advance about the barrier or patch depletion, ensuring that any behavioural changes did not reflect compliance with explicit instructions. Each patch was depleted after 10 collections, though this was not visually indicated and had to be discovered through trial-and-error. In addition, the risky level featured a suspenseful soundtrack, while the safe level had a calm one. In a pilot study, participants' subjective reports confirmed the elevated tension at the risky level. Both games are available online (see supplementary material).

### 2.2. Maze foraging game

**Figure 2.**
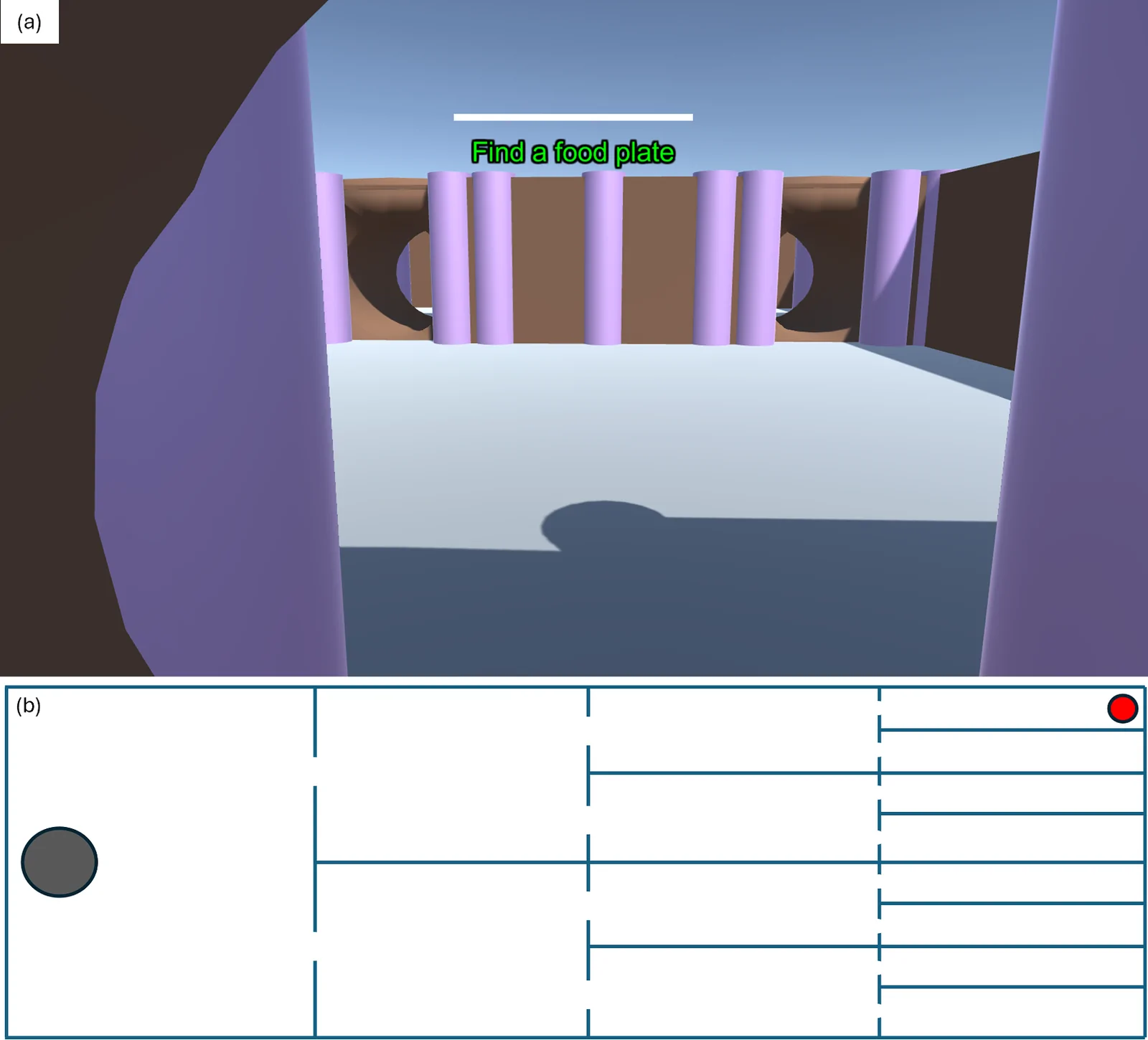
The maze game. (a) A snapshot from the game from a first-person view. A white time bar indicates the remaining time for the game. The snapshot shows a room in the game with two options the player faces. (b) A scheme of a 2 × 3 maze: the player has to choose between two options three times to reach the goal. The grey circle marks the start location, and the red circle represents the goal (a food plate), which was randomly placed in one of the last eight rooms of the maze.

**Figure 3.**
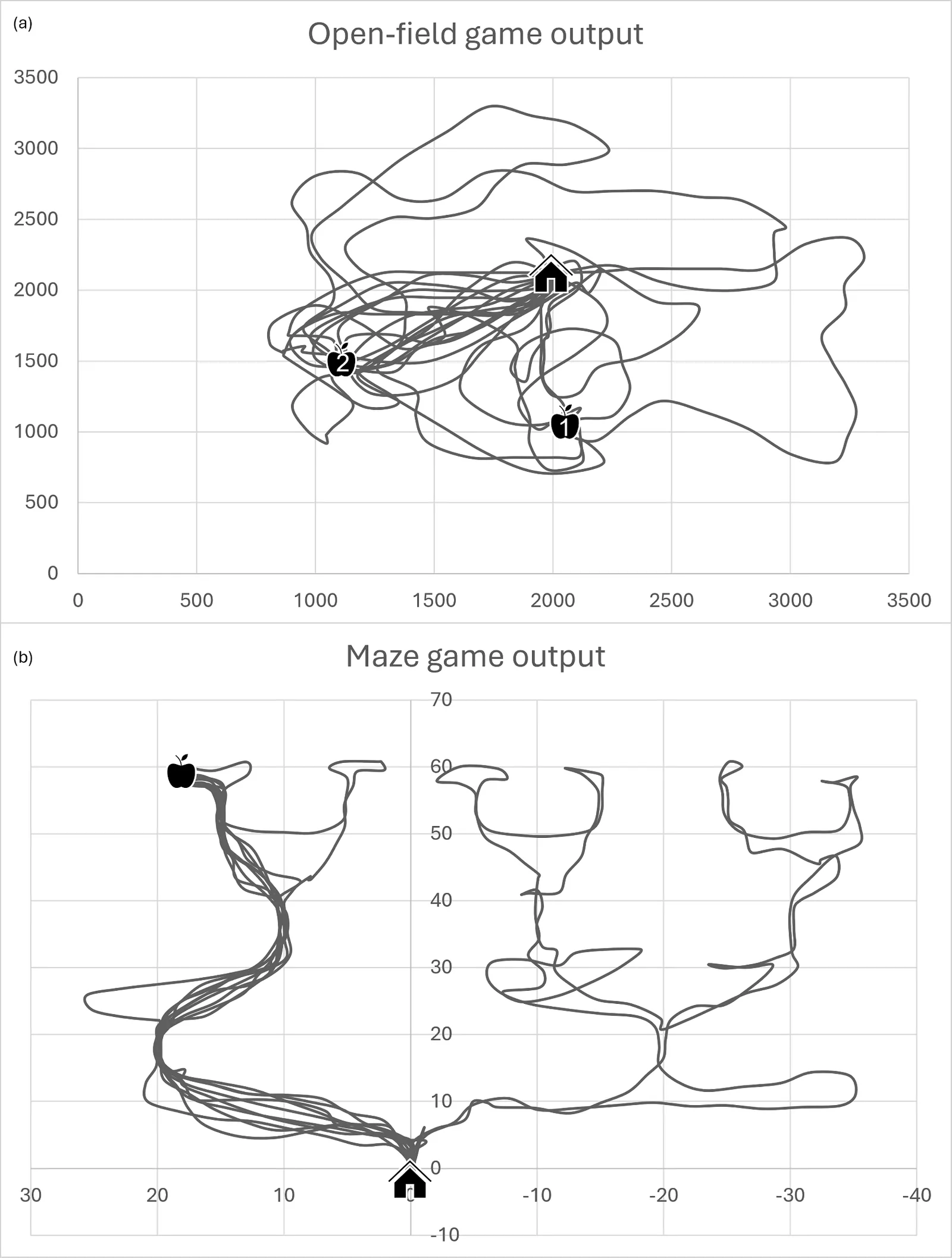
A connected scatter plot of output from both games for one of the participants. (a) Output of the open-field game: x- and y-axes are the player's coordinates in x and y, respectively. The grey line shows the full trajectory throughout the game. The house symbol shows the home location of the player, and apples show the locations of the food patches. Numbers on the apples indicate the patch arrival order in this example. (b) Output of the maze game: x- and y-axes are the player's coordinates in x and y, respectively. The grey line represents the player's path throughout the game. The house symbol shows the home location of the player, and the apple shows the location of the food patch.

We also developed  a three-dimensional, first-person game, where the player experiences the world through the eyes of the character, in Unity [60], and designed it to simulate a navigation task similar to laboratory experiments with desert ants [61–63]. The participants' field of view was 60°; the default in Unity. Participants navigated a binary-tree maze of three levels, where each cell splits into two, and only one path led to the goal (figure 2). The objective was to retrieve five green orbs from a fixed, hidden location and return each to the starting point. The goal remained constant throughout the game, requiring players to repeat the same route five times. Participants were given 15 minutes to complete the task; if they failed to locate the goal within this time, the game was terminated. This task assessed the participants' navigation ability and spatial learning and was used for comparison with performance in the open-field foraging game (figure 3).

### 2.3. Statistical analysis

All analyses and figures were produced using R 4.5.0 [64], with the packages ‘ggplot2’ for visualizations and ‘tidyr’ and ‘dplyr’ for data wrangling [65–67]. ‘lmertest’ was used for mixed-effect models, and ‘emmeans’ was used for marginal means comparisons [68,69].

For the open-field foraging game, we fitted four linear mixed-effects models, each targeting a different dependent variable. The models included 66 observations (33 participants × 2 levels). Fixed effects included risk level (risky or safe), level test order (risky first or safe first) and their interaction. Participant ID was included as a random effect to account for repeated measures.

The dependent variables were: (i) time spent hiding, defined as time spent inside the coordinates of any bush, which we expected to increase under predation risk. The game documents any movement next to bushes as hiding, but such values should naturally increase if the players linger longer within bushes. We applied a square-root transformation to the time spent hiding to improve the normality of residuals. (ii) Patch visits: number of times the player entered the food patch area, a measure of foraging effort independent of perceived score, expected to decrease under predation risk. (iii) Time to reach the first encountered food patch: a proxy for foraging and initial exploration, expected to decrease under predation risk. (iv) Time to reach the second patch after finding the first. This variable served as a proxy for the exploration–exploitation trade-off, as repeated visits made the first patch progressively more difficult to access owing to accumulating barriers. Shorter latencies to the second patch indicate earlier abandonment of the increasingly costly first patch and a greater exploratory tendency. Under predation risk, we predicted longer latencies to the second patch, reflecting reduced exploration. For each metric with a significant interaction, we made pairwise comparisons between every two groups by calculating the estimated marginal means of the model using the R package ‘emmeans’ [69].

For the maze game, we calculated a learning curve for each participant by segmenting the gameplay into five round trips (from the starting point to the food plate and back). We fitted both a linear model and an exponential mixed-effects model (as learning is typically exponential; [70]) with trip number (1–5) as the fixed effect, player ID as a random effect and trip time as the dependent variable. More complex models, such as exponential, will usually fit the data better simply because they include more parameters. To verify that the exponential form indeed provided a substantially better description of learning, we compared it against a simpler linear model using the Akaike information criterion (AIC). We applied a log transformation to the trip time to achieve normality of residuals.

Finally, we conducted cross-individual correlation analyses to test relationships between the performance of individuals in the open-field and maze games. We expected positive correlations, indicating innate spatial abilities or motivation. We used Pearson's correlation to assess the relationships between maze completion time and two out of the four open-field metrics: patch visits and time to reach the first encountered food patch, both serving as proxies for foraging intensity. In the maze game, maze completion time was used as a proxy for navigation ability.

Of the 35 participants, 33 (13 women and 20 men, aged 23–52 years) completed the open-field foraging game, of whom 31 completed both games. Sixteen participants played the risky level first, whereas 17 played the safe level first. In addition, as gender and age effects were not the main topic of this article and had only minor effects in a single model out of those described above, we report the analysis of these effects in the supplementary material.

## 3. Results

### 3.1. Open-field foraging game: predation risk and carryover effects:

**Figure 4.**
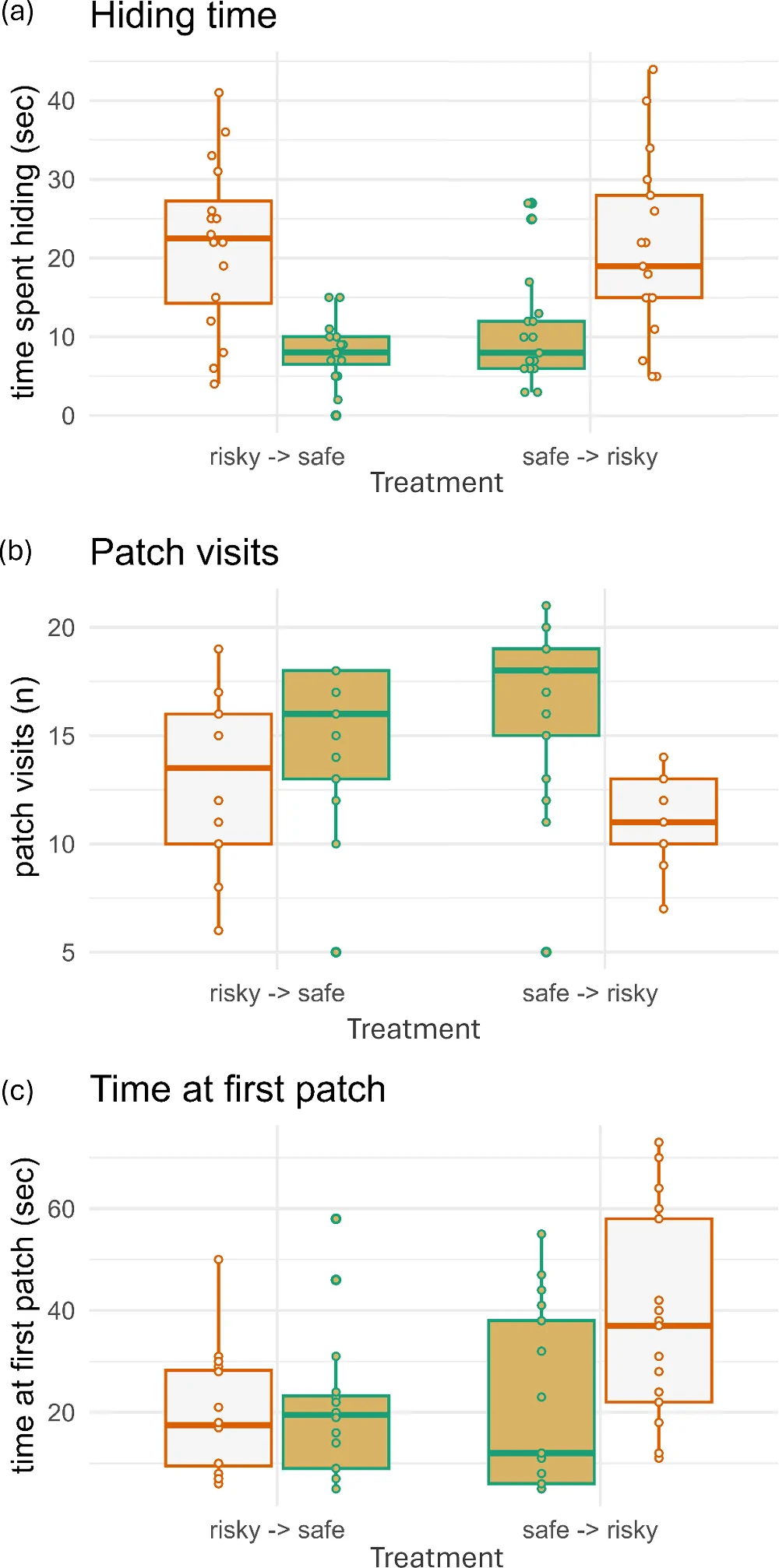
Box plots showing the three metrics that were measured in the open-field foraging game: (a) time spent hiding: time spent inside any bush. (b) Patch visits: number of times the player entered the food patch area. (c) Time to reach the first encountered food patch. Orange-white box plots represent the risky condition, and green-yellow ones represent the safe condition. Treatment labels on the x-axis indicate the order of exposure (risky to safe or safe to risky). Central lines indicate medians, boxes represent the interquartile range and circles represent individual participants.

*Patch visits:* patch visitation was significantly affected by predation risk and showed a significant interaction with test order (risk: *β* = 1.937, *t* = 2.189, *df* = 31, *p* = 0.036; risk × order: *β* = 3.180, *t* = 2.578, *df* = 31, *p* = 0.015), whereas test order alone was not significant (*β* = –1.823, *t* = –1.458, *df* = 49, *p* = 0.151). Post hoc comparisons revealed a clear carryover pattern (figure 4*b*). Participants who began in the safe context reduced patch visits by 5.12 visits when exposed to risk (*t* = 5.959, *df* = 31, *p* < 0.001). By contrast, participants who began under risk increased patch visits by 1.94 when subsequently tested in the safe context (*t* = –2.189, *df* = 31, *p* = 0.043), indicating attenuated sensitivity to the change in risk level. Between-individual differences accounted for 51% of the variance.

*Time to reach the first encountered food patch:* level test order and the interaction between risk and test order were significant (*β* = 19.048, *t* = 3.305, *df* = 54, *p* = 0.002; *β* = –17.511, *t* = –2.694, *df* = 31, *p* = 0.011), while risk level was not (*β* = 1.688, *t* = 0.362, *df* = 31, *p* = 0.720). Post hoc comparisons revealed a carryover pattern similar to that observed for patch visits (figure 4*c*). Participants who began in the safe context took 15.82 s longer to reach the first patch under risk (*t* = 3.496, *df* = 31, *p* = 0.001). By contrast, participants who began under risk showed no significant change when subsequently tested in the safe context (*t* = –0.362, *df* = 31, *p* = 0.725), indicating reduced sensitivity to context change following prior risk exposure. Between-individual differences accounted for 39% of the variance.

*Time spent hiding:* square-root-transformed time spent hiding increased significantly under predation risk (*β* = –1.807, *t* = -4.483, *df* = 62, *p* < 0.001; figure 4*a*). Neither the level test order nor the interaction between risk and order was significant (*β* = –0.104, 0.517, *t* = –0.262, *df* = 62, *p* = 0.794; *t* = 0.920, *df* = 62, *p* = 0.361).

*Time to reach the second patch after finding the first:* time to reach the second patch after finding the first was not significantly affected by risk level, test order or their interaction (all |*β*| ≤ 15.19, all |*t*| ≤ 1.42, all *p* ≥ 0.159). Contrary to our prediction, predation risk did not significantly influence switching to the alternative patch. Between-individual differences accounted for 36% of the total variance.

### 3.2. Maze game and learning dynamics

**Figure 5.**
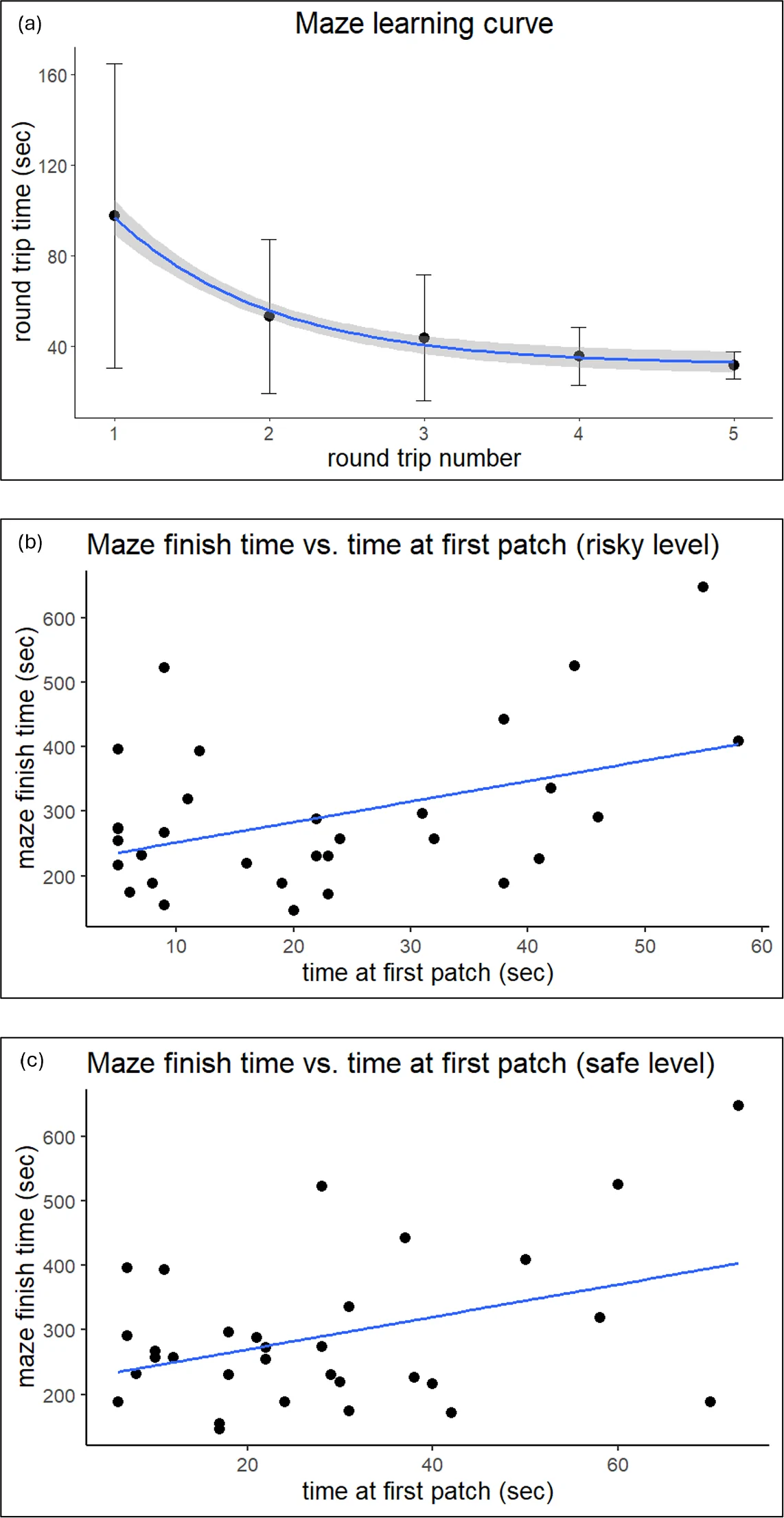
(a) Learning curve of participants in the maze game. Panels (b) and (c) show the relationship between maze completion time and time to reach the first food patch in the risky and safe conditions, respectively. Blue lines represent fitted regression lines. In panel (a), error bars indicate s.d., and the grey shaded area represents the 95% confidence interval around the fitted curve.

Trip duration declined significantly across successive round trips, consistent with spatial learning (*β* = –0.205, *t* = –9.556, *df* = 144, *p* < 0.001; figure 5*a*). The exponential model provided a substantially better fit than the linear model (ΔAIC = 1550), indicating a strongly negatively accelerated learning curve. Between-individual variance accounted for 25% of the total variance.

### 3.3. Cross-task correlations

*Maze finish time versus patch visits:* maze completion time was not significantly correlated with overall patch visitation in either context (*r* = –0.088, *p* = 0.638 for risky; *r* = –0.154, *p* = 0.409 for safe).

*Maze finish time versus time to reach the first encountered food patch:* maze completion time was positively correlated with latency to reach the first food patch in both contexts (*r* = 0.429, *p* = 0.016 for risky; *r* = 0.391, *p* = 0.030 for safe; figure 5*b*,*c*), indicating that individuals who were slower navigators in the maze were also slower to detect the first patch in the open-field task.

## 4. Discussion

We used ecologically structured computer games to examine how humans update and generalize information under changing predation risk. Our results highlight three main patterns. First, perceived predation risk altered behavioural allocation, consistent with risk-sensitive decision-making. Second, prior exposure to risk influenced subsequent performance after contextual change, indicating a behavioural carryover effect and suggesting incomplete updating. Third, individuals showed consistency in spatial performance across contexts and evidence of performance generalization across distinct tasks, which may reflect stable individual differences. This pattern should be viewed as preliminary, given that it is currently based on a limited number of cross-task associations. Learning curves exhibited the expected negatively accelerated form, consistent with experience-dependent improvement. Predation risk did not significantly alter switching behaviour between patches, suggesting that risk primarily affected overall foraging intensity rather than exploration.

A key finding was evidence for performance generalization across tasks. Individual performance was strongly correlated between the risky and safe contexts of the open-field task and moderately correlated with performance in the maze navigation task. This pattern suggests that spatial performance reflects a relatively stable individual characteristic rather than a purely context-specific strategy. Individuals who were efficient under predation risk also tended to perform well under safety and in a structurally distinct spatial task, indicating partial generalization of spatial ability across ecological contexts. Consistent individual differences in spatial behaviour have been documented across vertebrates [71], and spatial traits often show higher repeatability than commonly studied behavioural dimensions such as activity or boldness [72]. However, cross-task generalization is not universal [73,74], suggesting that task structure and cognitive demands constrain the extent of shared underlying mechanisms. Our findings, therefore, support the existence of stable individual differences in spatial learning ability while pointing to the importance of distinguishing domain-general navigation capacity from task-specific strategies.

Task sequence effects are a specific form of behavioural carryover, in which prior experience influences subsequent performance even after contextual change [75–77]. In our study, participants who experienced predation risk first continued to behave more cautiously in the subsequent safe context, despite explicit knowledge that the threat had been removed, while those who experienced safety first were more affected by the subsequent risk. This pattern suggests incomplete updating of threat-related representations and illustrates a potential cost of relying on outdated information [78]. In ecological terms, such persistence may lead to suboptimal foraging when environmental conditions shift [15,79].

Lingering effects of predation risk are well documented in animal systems, where exposure to predators can induce prolonged vigilance and delayed foraging even after the immediate threat has passed [80]. Psychological and neurobiological studies in humans demonstrate that learning of threats is rapid and resistant to extinction, such that fear responses may persist even after the danger is no longer present [81,82]. This persistence is often asymmetric, with threat-related associations enduring more strongly than safety-related ones [83]. Importantly, our results show asymmetry: prior exposure to safety altered subsequent behaviour in risk, whereas the reverse pattern was weaker. This pattern may have resulted from a stronger response to risk after a prior habituation to safe conditions and a weaker response to safety after habituating to risky conditions. Such asymmetry highlights the need to account for task sequence effects in experimental design, as behavioural responses may not be fully reset between contexts [84,85]. From both ecological and cognitive perspectives, threat representations may be updated more conservatively than safety representations, consistent with documented asymmetries in threat learning and risk-sensitive updating [86,87]. In ecological contexts, such conservative updating may be adaptive, as overestimating risk is typically less costly than underestimating it in natural environments [87].

Another possible interpretation of our results is as a combined effect of risk level and fatigue, both affecting the participants negatively. A subsequent neuroimaging study may aid in distinguishing between these two options: if activity in the fear-related amygdala region in the brain is stronger in the risky-after-safe trials than in the risky-first trials, the first option seems more likely, while a gradually reducing activity in focus-related areas across trials, like the frontal cortex, with no difference in amygdaloid function, will support the latter option.

Predation risk reduced foraging performance, consistent with extensive evidence in both animals and humans demonstrating the indirect behavioural effects of predators [5,8,22]. In our open-field paradigm, this reduction was expressed as increased hiding time at the expense of active foraging and reduced visitation of food patches, indicating a reallocation of behaviour towards safety. Prolonged shelter use under threat is well documented in ecological studies [88–90], and our results suggest that similar risk-sensitive allocation rules operate in human participants within a spatially structured task. In natural systems, predation risk often leads to earlier patch abandonment and higher giving-up densities [3,91]. Consistent with this, we observed reduced patch visitation under risk, indicating a shift in the balance between energy acquisition and perceived danger. Predation risk, however, did not alter patch-switching behaviour in our task. Patch value changed over time owing to increasing movement costs and depletion, and thus switching decisions were driven primarily by differences in patch value rather than by predation risk. This contrasts with systems in which predation risk is spatially heterogeneous, where differences in risk between patches can directly influence foraging decisions [92,93]. Experimental work further shows that such effects depend on how risk is distributed across foraging components, such as within patches versus during movement between them [13].

The negatively accelerated learning curve observed here is consistent with classic learning dynamics reported in both animals and humans [94,95]. Such curves are especially common in spatial learning tasks, including maze navigation in insects and rodents [61,96–98], and are typically interpreted as reflecting rapid early information acquisition followed by diminishing marginal gains. In ecological terms, this pattern suggests that individuals quickly extract coarse-grained structure from the environment and subsequently fine-tune their spatial representations. Future experiments could examine forgetting rates and interference effects, for example, by alternating between distinct spatial configurations, thereby testing how stable or context-dependent such representations are [63,99]. Examining the temporal persistence of carryover effects would also be a fruitful direction, for example, by introducing delayed retesting after different intervals, thereby testing the durability of such behavioural updating, including in virtual or game-based paradigms (e.g. [100]). Finally, future work should examine whether analogous behavioural measures show correlations among-individual performance across multiple ecological contexts, allowing a stronger test of performance generalization.

The study has several main limitations. Evidence for cross-task generalization is preliminary because it is based on a limited number of associations, and the minimal demographic information collected prevented a more thorough examination of the sources of individual variation. In addition, the observed order effects cannot be clearly distinguished from possible fatigue effects. Finally, the computer-game paradigm simplified ecological conditions: predation risk was symbolic, the environment was homogeneous and competitors were absent, limiting the ecological realism and generalizability of the findings but allowing precise control of decision dynamics.

In summary, our ecologically structured computer paradigms captured several principles of foraging theory: the trade-off between resource acquisition and predation avoidance, experience-dependent spatial learning, behavioural carryover effects across changing risk contexts and evidence of cross-task performance generalization. The absence of risk effects on patch switching suggests that threat primarily influenced global behavioural allocation rather than fine-grained strategic exploration. We view computer-based paradigms as complementary systems for testing ecological and cognitive principles under controlled conditions. Future work comparing humans and non-human animals within parallel task structures may clarify the extent to which shared mechanisms govern spatial learning and risk-sensitive behaviour.

## Supplementary Material



## Data Availability

The supplementary material consists of the dataset [101]. The game and R code used for the analysis appears on this link [102].
