# Supplementary material for: Using computer games to explore foraging-predation trade-offs and spatial learning in humans
Source: R Soc Open Sci. 2026 Sep 9;13(9):rsos260389. doi: 10.1098/rsos.260389 (PMC13561078; doi:10.1098/rsos.260389)
Supplement: Supplementary file 1 [file rsos.260389.zip › rsos.260389/rsos260389_si_001.docx]

**Supplementary Material for the manuscript entitled:**

**Using Computer Games to Explore Foraging-Predation Trade-offs and Spatial Learning in Humans**

By: Stav Subach, Arik Dorfman, Adi Bar, Tali Potudin, Aziz Subach, & Inon Scharf

We tested the additional effects of age and sex on the models discussed in the manuscript.

We added the main effects and interactions among all tested factors reported in the manuscript, and the sex and age of participants. We further removed the interactions in a stepwise manner and considered only the significant ones in the final models.

We have found significant effects only in the number-of-patch-visits model out of all tested models. The significant effects were of age and an interaction of test order, risk level, and sex. The results are summarized in the following table:

Table 1: number-of-patch-visits model results. 'X' designates interactions. Significant effects appear in bold.

|  | Estimate | SE | df | t | P |
| --- | --- | --- | --- | --- | --- |
| **(Intercept)** | **24.040** | **2.850** | **30.571** | **8.435** | **0.000** |
| risk: safe | 1.111 | 1.195 | 29.000 | 0.930 | 0.360 |
| **test order: safe first** | **-3.296** | **1.430** | **49.709** | **-2.304** | **0.025** |
| female | -2.897 | 1.604 | 49.706 | -1.806 | 0.077 |
| **age** | **-0.384** | **0.104** | **28.000** | **-3.711** | **0.001** |
| **risk: safe × test order: safe first** | **4.253** | **1.611** | **29.000** | **2.639** | **0.013** |
| risk: safe × female | 1.889 | 1.807 | 29.000 | 1.045 | 0.304 |
| **test order: safe first × female** | **5.627** | **2.361** | **48.180** | **2.383** | **0.021** |
| risk: safe × test order: safe first × female | -2.586 | 2.564 | 29.000 | -1.008 | 0.322 |

Age had a significant effect as well on this model; however, after removing the only participant who was above 30 (52 years old), the effect became not significant (estimated difference = -0.41 ± 0.24 SE, t = -1.74, P = 0.09).

Post-hoc comparisons of the estimated marginal means revealed no significant differences between males and females within any combination of risk level and test order (all P > 0.05). In the risky level, when the risky trial was presented first, males tended to exhibit higher values than females (estimated difference = 2.90 ± 1.60 SE, t = 1.81, P = 0.08), although this difference was not statistically significant. No sex differences were detected when the safe condition followed the risky trial (estimated difference = 1.01 ± 1.60 SE, t = 0.63, P = 0.53), when the risky condition followed the safe trial (estimated difference = −2.73 ± 1.72 SE, t = −1.58, P = 0.12), or when the safe condition followed the safe trial (estimated difference = −2.03 ± 1.72 SE, t = −1.18, P = 0.24).

To conclude, as for the number of visited patches, females seemed to react more to the change between the risky and safe conditions than males; however, as indicated by the post-hoc analysis, this effect was not significant. Overall, sex and age had limited or no effect on our results. However, the effects on the number of patch visits may indicate a larger response of females to changing risk. As this was not a principal question in our study, we can only point to a potentially interesting future lead by reporting this result.
